# How Cocaine Kills

**Published:** 1895-12

**Authors:** 


### How Cocaine Kills.

Maurel says that cocaine kills by first, dilating the small vessels; second, paralyzing the leucocytes; strong doses taken by the stomach act in this way. The toxic effect is proportional to the number of leucocytes paralyzed. Small doses, hypodermically, or in the veins, may act by paralyzing some cells which then become emboli. Large doses may be injected into the arteries without killing.—*Omaha Clinic.*

